# Fungal chromatin remodeler Isw1 modulates translation via regulating tRNA transcription

**DOI:** 10.1093/nar/gkaf225

**Published:** 2025-04-01

**Authors:** Jing Wang, Yueqi Zhang, Jingrui Wang, Liuqin Wang, Binshan Li, Shuang Liu, Xuepeng Sun, Zhonghua Ma

**Affiliations:** Department of Plant Pathology, College of Plant Protection, Southwest University, Chongqing 400715, China; National Key Laboratory of Rice Biological Breeding, Institute of Biotechnology, Key Laboratory of Molecular Biology of Crop Pathogens and Insects, Zhejiang University, 866 Yuhangtang Road, Hangzhou 310058, China; National Key Laboratory of Rice Biological Breeding, Institute of Biotechnology, Key Laboratory of Molecular Biology of Crop Pathogens and Insects, Zhejiang University, 866 Yuhangtang Road, Hangzhou 310058, China; National Key Laboratory of Rice Biological Breeding, Institute of Biotechnology, Key Laboratory of Molecular Biology of Crop Pathogens and Insects, Zhejiang University, 866 Yuhangtang Road, Hangzhou 310058, China; National Key Laboratory of Rice Biological Breeding, Institute of Biotechnology, Key Laboratory of Molecular Biology of Crop Pathogens and Insects, Zhejiang University, 866 Yuhangtang Road, Hangzhou 310058, China; Department of Plant Pathology, College of Plant Protection, Southwest University, Chongqing 400715, China; Department of Plant Pathology, College of Plant Protection, Southwest University, Chongqing 400715, China; Collaborative Innovation Center for Efficient and Green Production of Agriculture in Mountainous Areas of Zhejiang Province, College of Horticulture Science, Zhejiang A&F University, Hangzhou 311300, China; National Key Laboratory of Rice Biological Breeding, Institute of Biotechnology, Key Laboratory of Molecular Biology of Crop Pathogens and Insects, Zhejiang University, 866 Yuhangtang Road, Hangzhou 310058, China; Yazhouwan National Laboratory, Sanya 572025, China

## Abstract

Chromatin dynamics are essential for regulating DNA processes in response to environmental stimuli. Although ISWI-family enzymes are known to remodel chromatin by sliding nucleosomes in budding yeast, their functional roles and outputs in eukaryotes remain largely unknown. In this study, we investigated chromatin accessibility in the phytopathogenic fungus *Fusarium graminearum* treated with and without putrescine, a compound that rapidly induces the biosynthesis of the mycotoxin deoxynivalenol (DON). Putrescine globally alters chromatin accessibility, with the ATP-dependent chromatin remodeler FgIsw1 emerging as a key regulator. Unexpectedly, deletion of FgIsw1 did not affect the transcription of DON biosynthesis genes (Tri) but significantly disrupted transfer RNA (tRNA) transcription, leading to a dramatic decline in translation of DON biosynthesis enzymes. Mechanistically, FgIsw1 maintains nucleosome phasing in tRNA chromatin regions, ensuring efficient tRNA transcription. As a result, ΔFgIsw1 was unable to produce DON and lost its virulence on the host plant. These results highlight a novel role of chromatin remodelers in regulating protein translation through the control of tRNA transcription.

## Graphical Abstract

**Graphical Abstract:**
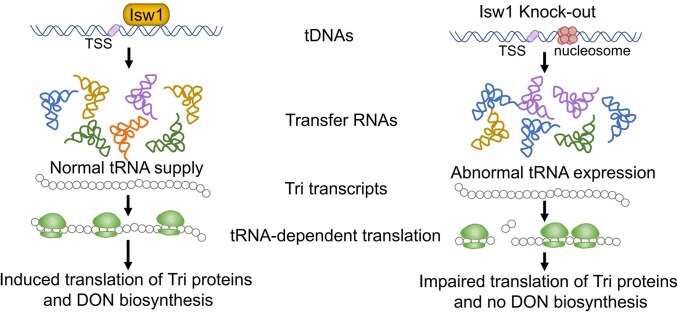

## Introduction

Eukaryotes have developed a series of intricate mechanisms to adapt to various environmental changes, with the reprogramming of chromatin playing a frontline role [1–3]. Upon the highly orchestrated structure of chromatin, reversible DNA methylation and covalent modifications on histone tail residues, including acetylation, phosphorylation, ubiquitination, or other post-translational modifications, are considered to mediate the stability of nucleosome octamer. Chromatin remodeling enzymes utilize the energy from ATP hydrolysis to reposition and rearrange nucleosomes, thus regulating gene transcription [4–6]. ISWI (imitation SWI/SNF), CHD (chromodomain helicase DNA-binding protein), SWI/SNF (switch/sucrose nonfermentable), and INO80 (inositol-requiring mutant 80) are well known four subfamilies of ATP-dependent chromatin remodeling enzymes [7, 8] that are responsible for nucleosome assembly and organization, chromatin access, and nucleosome editing. The ISWI subfamily, for example, organizes nucleosomes to regulate chromatin accessibility and gene expression [9, 10]. In budding yeast, ISWI ATPases Isw1 and Isw2 form three distinct complexes and are involved in positioning nucleosomal arrays at a variety of gene regulatory regions [11, 12]. Moreover, ISW2 is also recruited to specific regions by the Ume6 repressor [13]. In *Drosophila*, there is only one ISWI ATPase that is assembled into three distinct complexes: ACF (ATP-dependent chromatin assembly and remodeling factor), NURF (nucleosome remodeling factor), and CHRAC (chromatin accessibility complex) [14–16]. Similar complexes have been found in *Neurospora crassa* [17], and a triple mutant lacking three ISW accessory factors or disrupting multiple ISW complexes lead to global up-regulation of PRC2 (polycomb complex 2) target genes and altered H3K27 methylation pattern [16]. These findings indicate that ISWI complexes may modulate gene expression by affecting chromatin organization [18], though the detailed mechanisms and functional outputs remain largely unexplored.

*Fusarium graminearum* is one of the most important causal agents of *Fusarium* head blight (FHB) on wheat, barley, and other cereal crops worldwide. Recent severe outbreaks of FHB have resulted in substantial yield losses, economic damage, and increased concerns about mycotoxins contamination, particularly deoxynivalenol (DON) [19]. DON is a secondary metabolite (SM) of *F. graminearum*, and its biosynthetic enzymes are encoded by 15 Tri genes. Previous studies have shown that transcription and translation of Tri genes are rapidly and highly induced by putrescine serving as the solely nitrogen source in toxin biosynthesis inducing (TBI) medium [20, 21]. This provides an ideal model for investigating regulatory mechanisms of transcription and translation machinery in pathogenic fungi.

Previous studies have shown that histone acetylation and methylation play crucial roles in regulating expression of Tri genes. However, the impacts of other epigenetic factors in DON modulation are lacking [20]. Chromatin remodelers have been appeared at the forefront for understanding the regulation of gene expression [22]. Moreover, many genes involved in fungal SMs are arranged in clusters that may be significantly affected by epigenetic modifications [23]. Therefore, characterizing functions of chromatin remodelers in the regulation of DON biosynthesis can offer a valuable opportunity to enhance our understanding outputs of chromatin remodelers.

In current study, we identified and characterized ATP-dependent chromatin remodeling complex ISWI by taking the advantage of DON induction system in *F. graminearum*. By combining high-throughput sequencing with genetic and biochemical assays, we found that the chromatin remodeling enzyme FgIsw1 targets chromatin segments of tRNA genes and modulated their expression in response to putrescine treatment. This fluctuation in the tRNA pool alters translation efficiency, resulting in suppressed translation of DON biosynthesis enzymes and blocking its biosynthesis in the FgIsw1 mutant. Overall, this study provides a conceptual framework for the role of chromatin remodeling by ISWI in regulating translation via tRNA transcription, enriching our understanding of the roles of ISWI chromatin remodeler beyond transcription activation.

## Materials and methods

### Fungal strains and culture conditions

The *F. graminearum* strain PH-1 (NRRL31084) was used as the wild-type control strain for transformants generation in this study. The potato dextrose agar (PDA) (200 g potato, 10 g D-Glucose, 10 g agar, and 1 l water) was used as an ordinary for the growth and growth rate examinations of wild type and transformant strains, together with complete medium (CM) [24] and minimal medium (MM) [25].

For deoxynivalenol induction, newly grown and collected mycelia were incubated in TBI (30 g sucrose, 1 g KH_2_PO_4_, 0.5 g MgSO_4_•7 H_2_O, 0.5 g KCl, 0.01 g FeSO_4_•7H_2_O, 1.47 g putrescine hydrochloride, 200 μl trace element, and 1 l water, pH 4.5) [26], for ATAC-seq, RNA-seq, confocal, and WB, mycelia of each strain were incubated in TBI broth in a shaker at 28°C for 40 h, for DON production determination, the incubation should be extended to 7 days.

### Strains construction

Gene deletion mutants of *F. graminearum* were constructed using polyethylene glycol (PEG) mediated protoplast transformation method [27]. The full-length and truncated FgIsw1 complement strains were also constructed in the PEG-mediated protoplast transformation. Briefly, a full-length or truncated ORF segment of FgIsw1 was amplified from the genome of wild-type PH-1, extracted, and fused extracted with GFP/RFP/mCherry and resistance (hygromycin B, geneticin, or nourseothricin) coding genes amplified from vectors with multi-segment nested PCR, then the third cycle PCR product was re-transformed into the corresponding mutant. Taking the DEXDc-truncated complement strain FgIsw1-CD as an example, the ORF of FgIsw1 was divided into two sections and amplified from the DNA extracted from PH-1: one from the upstream 1000 bp to the base right before where the DEXDc domain start, the other is precisely started from the base followed-by the DEXDc motif and ended before the termination codon. At the same time, the GFP and geneticin coding genes, and the downstream 1000 bp segment of FgIsw1 coding gene were amplified and recovered. These six segments were fused with nested PCR next. Finally, after verification of the long segment of “upstream-FgIsw1-CD-GFP-geneticin-downstream,” it was re-transformed into the protoplast of ΔFgIsw1 strain, other strains were constructed in a similar way.

### ATAC-seq library preparation, differential accessibility, and nucleosome map of tRNA genes

Approximately 5 × 10^4^ nuclei were extracted directly from the wild-type PH-1 or ΔFgIsw1 under specific growth conditions (TBI or TBI without putrescine). ATAC-seq libraries were generated according to the instructions of the TruePrep Index Kit V4 for illumina (Vazyme Biotech Co., Ltd, Nanjing, China). Peaks were called on the merged set of all ATAC-seq reads using MASC2 [28] and filtered to remove putative copy number altered regions. The number of reads/peaks was determined for each sample using bedtools multicov, and the relative sequencing depth was estimated using a set of “housekeeping” peaks at transcription start sites (TSSs) of genes that were uniformly expressed in this fungus. Differential accessibility was assessed using DESeq2 [29]. Unless otherwise stated, regions were called differentially accessible if the absolute value of the log2 fold change was 0.5 at an FDR <0.1. Visualizations of insertion tracks were smoothed by 150-bp sliding windows (20-bp step size). To obtain the nucleosome positional information, we merged replicates for each condition and ran nucleoATAC [30] with default parameters on a combination of all peaks in all samples. The experiment was repeated twice independently.

### ChIP-seq

Chromatin immunoprecipitation (ChIP) was performed as previous describe with additional modifications [31]. Briefly, fresh mycelia were cross-linked with 1% formaldehyde for 20 min and then stopped with 125 mM glycine for 10 min, which were ground into powder with liquid nitrogen immediately. Next, the mycelial powder was re-suspended in lysis buffer (250 mM, HEPES pH 7.5, 150 mM NaCl, 1 mM Ethylene Diamine Tetraacetic Acid (EDTA), 1% Triton, 0.1% DeoxyCholate, and 10 mM Dithiothreitol (DTT)) and protease inhibitor (Sangon Co., Shanghai, China). DNAs were sheared into ∼300 bp fragments with the high amplitude (30 s’ on and 30 s’ off, 18 cycles, Qsonica*sonicator, Q125, Branson, USA). The supernatant was diluted with 10× ChIP dilution buffer (1.1% Triton X-100, 1.2 mM EDTA, 16.7 mM Tris–HCl, pH 8.0, and 167 mM NaCl) after centrifugation. Immunoprecipitation was performed using monoclonal anti-GFP ab290 (Abcam, Cambridge, UK; 1:500 dilution) antibody together with the protein A agarose (sc-2001, Santa Cruz, CA, USA) respectively. After washed orderly by low salt wash buffer (150 mM NaCl, 20 mM Tris–HCl, pH 8.0, 0.2% SDS, 0.5% Triton X-100, 2 mM EDTA, one time), high salt wash buffer (500 mM NaCl, 20 mM Tris–HCl, pH 8.0, 0.2% SDS, 0.5% Triton X-100, 2 mM EDTA, one time), LiCl wash buffer (one time), TE buffer (100 mM Tris–HCl, pH 8.0, 10 mM EDTA, two times), the admixture of DNA-protein was eluted from beads with elution buffer (1% Sodium dodecyl sulfate (SDS), 0.1 M NaHCO_3_). After salt reversing and proteinase K digestion, the DNAs were precipitated with isopropanol, washed with ethanol, and dissolved in water. Finally, ChIP-enriched DNA was sent for sequencing on an Illumina HiSeq 250 (Novogene Bio., Tianjin, China).

The ChIP-seq data analysis was carried out as follows: Trimmomatic (version 0.36) was used to filter out low-quality reads [32]. Clean reads were mapped to the *F. graminearum* genome by Bwa (version 0.7.15) [33]. Samtools (version 1.3.1) was used to remove potential PCR duplicates [34]. MACS2 software (version 2.1.1.20160309) was used to call peaks by default parameters (bandwidth, 300 bp; model fold, 5, 50; q value, 0.05). Peaks were assigned to the gene closest to their midpoint, particularly if it was located near the gene’s TSS [35]. For peak annotation, the coding sequence was defined as gene body, the 1 Kb upstream the messenger RNA (mRNA) region was defined as promoter, the 1 Kb downstream the mRNA region was defined as downstream, regions >1 Kb upstream or downstream the mRNA was defined as distal intergenic regions.

### RNA-seq analysis

Each strain was inoculated and incubated with agitation (180 rpm) for 24 h at 25°C in 200 ml CM or 40 h at 28°C TBI liquid medium, then total RNAs were isolated from the harvested mycelia with the TRIzol Reagent (Life Technologies, US) and shipped to Novogene Bio. (Tianjin, China) for library construction and sequencing with Illumina Hiseq 150 sequencer. Three replicates for each strain under each condition were sequenced. For each sample, 35–50 M clean RNA-seq reads were obtained and mapped to the *F. graminearum* PH-1 genome sequence using Hisat2.

### MNase-qPCR analyses

The MNase assay was conducted according to a previously reported protocol [36] with additional modifications. To isolate fungal nuclei, 2 g fresh liquid nitrogen frozen mycelia were grounded to a fine powder with pre-cooled mortar and pestle. The resulting mycelial powder collected in a pre-cooled 50-ml reaction tube and resuspended in 10 ml nuclei isolation buffer (NIB, 0.44 M sucrose, 1.25% Ficoll, 2.5% dextran T40, 20 mM HEPES-KOH, pH 7.4, 10 mM MgCl_2_, 0.5% Triton X-100, 5 mM DTT). The suspension was filtered through two layers of Miracloth (Merck Millipore, Catalogue No. 475855). After 15 min centrifuge at 4°C, 2500× *g*, the pellet was resuspended in 1 ml of NIB and centrifuged for 10 min at 4°C, 2500× *g* and repeat this step for at least for three times to wash the nuclei. Then the pellet was washed with 1 ml of MNase reaction buffer (MRB, 20 mM Tris–HCl pH 8, 5 mM NaCl, 2.5 mM CaCl_2_) and resuspended in 660 μl of MRB. There, 160 μl of nucleus suspension was transferred to a new 1.5-ml reaction tube and added up to 500 μl with MRB as undigested control, the remaining 500 μl of solution was divided into several MNase-treated samples and digested with MNase (Sigma, N3755) at 37°C for 8 min (70–80 ng DNA per sample, the final concentration of MNase was 0.01–0.02 units/μl). Then the digestion was terminated by adding 50 μl of Stop buffer (100 mM EDTA and 100 mM Ethylene Glycol Tetraacetic Acid (EGTA)), 50 μl 10% SDS, and 40 μg proteinase K and incubated at 60°C for 1 h. After overnight RNase treatment at 4°C. Genomic DNA (gDNA) of each sample and control was extracted with phenol–chloroform–isoamyl alcohol and precipitate with 100% ethanol. The resulting gDNA of each sample was used as template in the following qPCR analyses for nucleosome occupancy determination. Tiled oligonucleotides spanning the region of 200 bp upstream and 200 bp downstream of the TSS of each tDNA locus were designed and used (Supplementary Table S4). The experiment was repeated three times independently.

### EMSA assays

The complementary DNA (cDNA) segments encoding indicating proteins were amplified and cloned into pET21b(+) vector to generate 6× His-tagged proteins. The resulting construct was transformed into the *Escherichia coli* strain BL21 (DE3) after verifying the cDNA sequence. The recombinant 6× His-tagged proteins were purified and dialyzed into 1× DNA binding buffer (DBB, 10 mM Tris–HCl, pH 7.5, 0.05 M NaCl, 1 mM DTT, 1 mM EDTA, 5% glycerol). Single-stranded tDNA segments were synthesized from Sangon Biotech (Shanghai, China) Co., Ltd and annealed. For electrophoretic mobility shift assay (EMSA), reaction mixtures containing purified 6× His-tagged protein and tDNA in 1× DBB were incubated for 20 min at 25°C. Then reactions were electrophoresed on 1.2% agarose gel in 0.5× TAE for 45 min at 80 V under low temperature. Signals were detected in J3-3000 imaging system after dying DNA dye ethidium bromide for 15 min. The experiment was conducted independently three times.

### Label-free LC-MS-based proteomics

Total protein was extracted from fresh harvested mycelia PH-1 or ΔFgIsw1 growth in CM or TBI grown. After trypsin digestion, protein samples were sent to nano LC-MS/MS analysis (BiotechPack Scientific, Beijing, China). The raw MS files were analyzed and searched against target protein database (https://www.uniprot.org/) based on the species of the samples using MaxQuant [37]. Three replicates for each strain under each condition were sequenced. For each sample, the peptide solution was transferred to mass spectrometry analysis for 2 h.

### Toxisome induction, DON production, and virulence evaluation

To observe toxisome formation in PH-1 and derived mutants, Tri1-GFP labeled PH-1 and other strains were cultured in TBI liquid medium for 40 h before observation with a confocal microscope, the expression of Tri1-GFP was also detected with western blotting using an anti-GFP antibody. To quantify DON producti, each strain was grown in CM broth for 24 h at first, then the newly mashed mycelia were transferred into TBI liquid medium, and DON was extracted from each strain after incubation for 7 days. The cell-free supernatant was filtered and passed through a SampliQ Amino (NH2) solid phase extraction column (Agilent Technologies), and 4 ml of the purified extraction was evaporated to dryness under a nitrogen stream. The residue was dissolved in 1 ml methanol: water (40:60, *v*/*v*), followed by centrifugation at 10 000 rpm and subsequently analyzed by LC-MS/MS [38].

Given that the mutants were defective for conidiati, 0.1 mg fresh mycelia of each strain were used to inoculate a middle spikelet of flowering wheat head. Twenty individual wheat heads were inoculated for each tested strain. Then the inoculated wheats were kept in a humility of 90%–100% for 15 days, representative images were taken and the disease indexes were calculated, experiments were repeated three times.

## Results

### Genomic localization of Isw1 in *F. graminearum*

**Figure 1.**
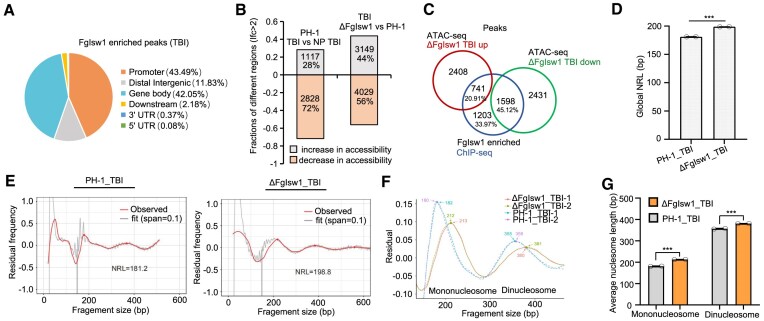
FgIsw1 regulates chromatin accessibility and nucleosome pattern. (**A**) Distribution of FgIsw1 enriched peaks on DNA regions detected by ChIP-seq. (**B**) Chromatin accessibility change caused by putrescine inducing or FgIsw1 depletion detected by ATAC-seq analysis. (**C**) Venn diagram showing overlap between ChIP-seq detected FgIsw1-enriched peaks and ATAC-seq captured accessibility increased or decreased chromatin regions. (**D**) Bar graphs of the global nucleosome repeat length (NRL) of ΔFgIsw1 versus the wild-type PH-1 grown in TBI based on the ATAC-seq data (*n* = 2). (**E**) Nucleosome phasing graph showing changes in the NRL among ΔFgIsw1 and PH-1 upon putrescine induction. ***P < .01. (**F**) Average fragment size of mononucleosomes and dinucleosomes of PH-1 and ΔFgIsw1 grown in TBI. (**G**) A bar graph showing the average size of nucleosomes in TBI cultured PH-1 and ΔFgIsw1 in the figure F (*n* = 2).

FgIsw1 contains conserved chromatin binding motifs: two SANT domains, and two helicase ATP-binding motifs: DEXDc and HELICc (Supplementary Fig. S1A and B). To facilitate the analysis of endogenous Isw1 in *F. graminearum*, we incorporated an *in-situ* GFP epitope-tagged FgIsw1 strain (named ΔFgIsw1::FgIsw1-GFP, where the FgIsw1-GFP fragment was transformed into the ΔFgIsw1 mutant). Then, we performed ChIP combined with deep sequencing (ChIP-seq) using the anti-GFP antibody to map localization of FgIsw1 throughout the genome. Mycelia grown in the TBI broth were cross-linked with formaldehyde, the FgIsw1 enriched DNA segments were precipitated, isolated, and subjected to high-throughput sequencing. A total of 3542 peaks were identified in FgIsw1-GFP precipitates, corresponding to 3079 genes; and these peaks were distributed relatively evenly across the four chromosomes (Supplementary Table S1). Notably, 42.05% of these peaks overlapped within gene bodies, which was comparable to these peaks overlapped within promoter regions (43.49%). In contrast, fewer peaks were associated with intergenic regions (11.83%) and even fewer with downstream regions (2.18%), 5′ UTRs (0.08%), and 3′ UTRs (0.37%) (Fig. 1A). These results suggest that FgIsw1 predominantly binds gene bodies and promoters, with minimal association with other gene regions.

### Lack of Isw1 affects chromatin architecture

Previous studies have demonstrated that the ATP-dependent chromatin remodeling enzyme ISW1 can slide nucleosomes along DNA [9, 39], with yeast Isw1 being well-characterized for its role in nucleosome phasing and spacing regulation [40, 41]. To explore how FgIsw1 affects chromatin accessibility, we conducted ATAC-seq (assay for targeting accessible chromatin with high-throughput sequencing) in *F. graminearum*. Taking advantages of the induced expression of TRI genes of *F. graminearum* in TBI medium (trichothecene biosynthesis inducing broth in which putrescine serves as the sole nitrogen source) [21], we compared the chromatin accessibility of ΔFgIsw1 with the wild-type PH-1 cultured in TBI, and in non-putrescine containing TBI (NP-TBI). This analysis identified that PH-1 had 1117 peaks gained and 2828 peaks lost in the TBI compared to those in the NP-TBI (Fig. 1B). In contrast, ΔFgIsw1 cultured in the TBI showed 3149 peaks gained and 4029 peaks lost, respectively, as compared to those of PH-1 under the same condition (Fig. 1B). To further elucidate the role of FgIsw1 in chromatin accessibility regulation, we compared FgIsw1-enriched peaks from ChIP-seq with accessible peaks identified by ATAC-seq. Notably, 45.12% FgIsw1 enriched peaks were less accessible in ΔFgIsw1 than in the wild-type PH-1 in TBI, while 20.91% peaks were more accessible. Approximately one-third (33.97%) of FgIsw1 enriched peaks showed no significant change in accessibility or were undetected (Fig. 1C). Overlapping genes corresponding to these peaks in each group displayed consistent trends (Supplementary Fig. S1C). Taken all, FgIsw1 directly binds to chromatin and likely modulates chromatin accessibility.

To determine whether these accessibility changes were linked to chromatin architecture, we calculated average nucleosome repeat length (NRL) using the ATAC-seq data, employing a local regression model with two smoothing parameters [42]. This analysis revealed that an increased NRL in ΔFgIsw1 compared to the wild-type PH-1 grown in TBI. Specifically, PH-1 exhibited an NRL of ∼181 bp, while ΔFgIsw1 displayed an NRL of ∼199 bp (Fig. 1D and E). Although the depth of our ATAC-seq sequencing was insufficient to measure NRL at individual genomic features, the data suggest that FgIsw1 regulates global NRL. In addition, based on the ATAC-seq data, nucleosome phasing analysis indicated that ΔFgIsw1 exhibited broader and lower-amplitude peaks on gene bodies in both mono- and di-nucleosomes, indicating weaker nucleosome phasing (Fig. 1F and G). These findings collectively indicate that FgIsw1 regulates chromatin accessibility and is crucial for proper nucleosome spacing.

### Isw1 targets tRNA genes and modulates the tRNA pool

**Figure 2.**
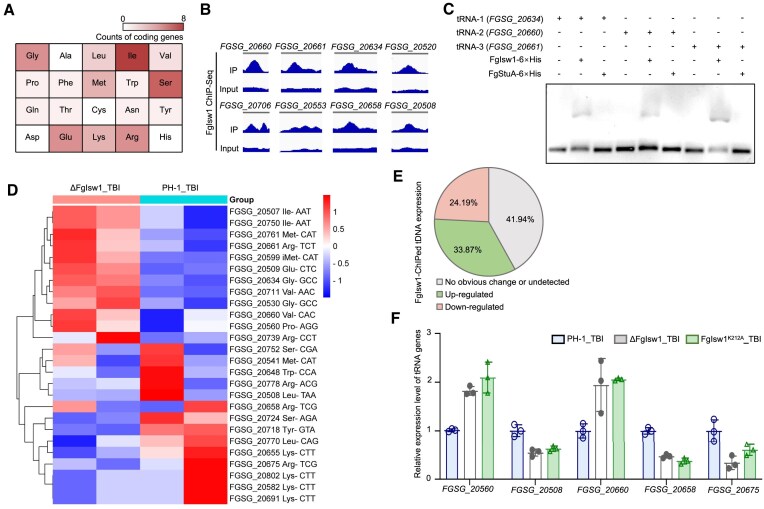
FgIsw1 targets tRNA genes and regulates the tRNA expression. (**A**) Heatmap of FgIsw1 enriched tRNA genes detected by ChIP-seq. (**B**) IGV browser tracks showing example tDNA peaks of FgIsw1 obtained in ChIP-seq experiment. (**C**) EMSA shows that FgIsw1 bound the tDNAs locus *FGSG_20634*, *FGSG_20660*, and *FGSG_20661*. In contrast, the transcription factor FgStuA, which was used as a negative control due to its known ability to bind target gene promoters and regulate their expression, did not bind these loci. (**D**) FgIsw1 bound tRNA genes and modulated their transcription. Pie chart of expression change of FgIsw1 enriched tRNA genes caused by FgIsw1 deletion. (**E**) Expression patterns of FgIsw1 enriched tDNAs in putrescine induced PH-1 and ΔFgIsw1. (**F**) A bar graph showing the levels of several FgIsw1 enriched tDNAs in putrescine induced PH-1 (PH-1_TBI), ΔFgIsw1, and the ATPase inactivated mutant strain FgIsw1^K212A^, as detected by RT-qPCR (*n* = 3).

The ChIP-seq analysis of FgIsw1 identified five peaks corresponding to ribosomal RNA (rRNA) gene, and 65 peaks associated with 62 transfer RNA (tRNA) genes (Fig. 2A and B, and Supplementary Table S1), suggesting that 19.94% tRNA genes might be targets by FgIsw1. To verify FgIsw1 binding activity to the tDNA, we performed EMSA using purified FgIsw1 and synthesized tDNA segments. The FgIsw1-6× His protein was expressed in *E. coli* and purified. Several single-stranded tDNA segments, including *FGSG_20634*, *FGSG_20660*, and *FGSG_20661*, were annealed with their reverse complementary sequences and co-incubated with FgIsw1-6× His. These tDNA segments were selected based on their high binding potential as indicated by the ChIP-seq data. The EMSA results demonstrated that FgIsw1 directly bound these tDNA segments, whereas the control 6× His-tagged transcription factor FgStuA did not (Fig. 2C).

To further figure out the effect of FgIsw1 on the expression of tRNA and protein-coding genes, we conducted tRNA sequencing and transcriptomic analysis comparing wild-type PH-1 and ΔFgIsw1 grown in TBI. The tRNA-chain-specific tRNA sequencing was used to determine the tRNA gene expression levels, while the ploy A chain enriched transcriptomic sequencing was used for estimating expression of the protein-coding genes. The PH-1 and ΔFgIsw1 grown in NP-TBI served as references (Supplementary Fig. S2A). The reliability of the RNA-seq data was confirmed by selecting 10 random genes for validation (Supplementary Fig. S2B). Interestingly, tRNA-seq revealed that 58.06% of FgIsw1-enriched tRNAs showed differential expression in ΔFgIsw1 compared to PH-1 grown in TBI, with 33.87% upregulated and 24.19% downregulated (Fig. 2D and E, and Supplementary Table S2). Quantitative real-time PCR (RT-qPCR) further confirmed these transcriptional changes in FgIsw1 target tRNAs (Fig. 2F). In contrast, only 4.79% of FgIsw1-ChIPed protein-coding genes were up-regulated, 4.51% were down-regulated, whereas 90.70% showed no significant transcriptional differences between ΔFgIsw1 and PH-1 or undetected in the transcriptomic analyzing (Supplementary Fig. S2C and Supplementary Table S3). Moreover, the expression of the trichothecene biosynthesis genes and the nonribosomal peptide synthetase gene (NRPS8) in PH-1 and ΔFgIsw1 were comparable (Supplementary Tables S3 and S4), even though ΔFgIsw1 exhibited changes in the expression of ∼2000 protein-coding genes relative to PH-1 grown in TBI (Supplementary Fig. S3). Overall, these results indicate that FgIsw1 directly binds tDNAs and plays a key role in regulating their transcription.

### Isw1 regulates nucleosome occupancy of tDNAs

**Figure 3.**
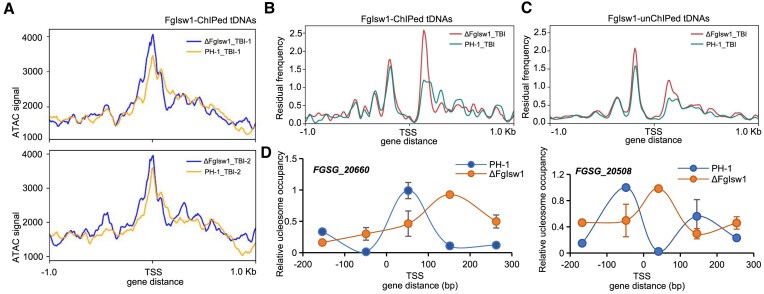
FgIsw1 regulates nucleosome occupancy of tDNAs. (**A**) Comparisons in chromatin accessibility of FgIsw1 enriched tRNA genes in TBI cultured wild-type PH-1 (*n* = 2: PH-1-1 and PH-1-2) and ΔFgIsw1 (*n* = 2: ΔFgIsw1-1 and ΔFgIsw1-2) plotted based on ATAC-seq data. (**B**) Comparisons in chromatin accessibility of FgIsw1 enriched tDNAs in TBI cultured wild-type PH-1 (*n* = 2: PH-1-1 and PH-1-2) and ΔFgIsw1 (*n* = 2: ΔFgIsw1-1 and ΔFgIsw1-2) plotted based on ATAC-seq data. (**C**) Comparisons in chromatin accessibility of FgIsw1 un-enriched tDNAs in TBI cultured wild-type PH-1 (*n* = 2: PH-1-1 and PH-1-2) and ΔFgIsw1 (*n* = 2: ΔFgIsw1-1 and ΔFgIsw1-2) plotted based on ATAC-seq data. (**D**) Nucleosome occupancy at tDNA locus *FGSG_20660* and *FGSG_20508*. Nucleosome occupancy in TBI cultured PH-1 or ΔFgIsw1 was determined by MNase treatment followed by RT-qPCR analysis. The signal was plotted ∼−200 to + 250 bp from the TSS. Data presented are the mean ± standard errors from three repeated experiments.

Analysis of the ATAC-seq data revealed that the FgIsw1 target tDNAs were significantly less accessible in ΔFgIsw1 compared to the wild-type PH-1 (Fig. 3A and Supplementary Fig. S4A). We plotted the normalized nucleosome occupancy centered on these FgIsw1 enriched tDNAs (Fig. 3B), and found that nucleosome phasing around these tDNA segments was markedly disrupted in ΔFgIsw1 compared to the wild-type PH-1. This disruption was particularly evident for the first nucleosome downstream of the TSS (Fig. 3B and Supplementary Fig. S4B). In contrast, analysis of FgIsw1-unenriched tDNAs, using data from the GtRNAdb online database (https://gtrnadb.ucsc.edu/GtRNAdb2/genomes/eukaryota/Fusa_gram_PH_1_NRRL_31084/Fusa_gram_PH_1_NRRL_31084-summary.html), showed no significant changes in nucleosome occupancy following FgIsw1 deletion (Fig. 3C). Similarly, nucleosome occupancy in the coding regions of FgIsw1-enriched protein-coding genes remained unchanged between ΔFgIsw1 and the wild-type PH-1 grown in TBI or NP-TBI (Supplementary Fig. S4C).

To further assess FgIsw1’s role in tDNA nucleosome occupancy, we performed MNase-qPCR (micrococcal nuclease-quantitative PCR) on specific chromatin segments. We targeted two tDNAs, *FGSG_20660* and *FGSG_20508*, which encode tRNA-Val Guanine Uracil Guanine (GUG) and tRNA-Lue Uracil Uracil Adenine (UUA), respectively. Mycelia from wild-type PH-1 and ΔFgIsw1 strains were prepared as in the ATAC-seq, the nuclei were extracted and subjected to MNase digestion, followed by gDNA extraction and RT-qPCR analysis. The tiled primer sets used for this experiment are listed in Supplementary Table S5. We analyzed the nucleosome occupancy from upstream 200 bp (−200 bp) to downstream 250 bp (250 bp) to the TSS of *FGSG_20660* or *FGSG_20508* in ΔFgIsw1 and the wild-type PH-1 grown in TBI. The tDNA region of *FGSG_20660* and *FGSG_20508* were 90 and 104 bp in length, respectively. Since tDNAs are typically <150 bp and generally be considered nucleosome-free [43], we focused on whether the presence of nucleosomes in these tDNA regions. The results in Fig. 3D show lower nucleosomal signals of *FGSG_20660* (0–90 bp) and higher nucleosomal signals of *FGSG_20508* (0–104 bp) in ΔFgIsw1 compared to PH-1, indicating that, although these tDNA regions are normally nucleosome-free, FgIsw1 depletion alters nucleosomal occupancy. Overall, these findings suggest that FgIsw1 is essential for maintaining regular nucleosome occupancy in its target tDNAs.

### FgIsw1 controls protein biosynthesis via tRNA transcription

**Figure 4.**
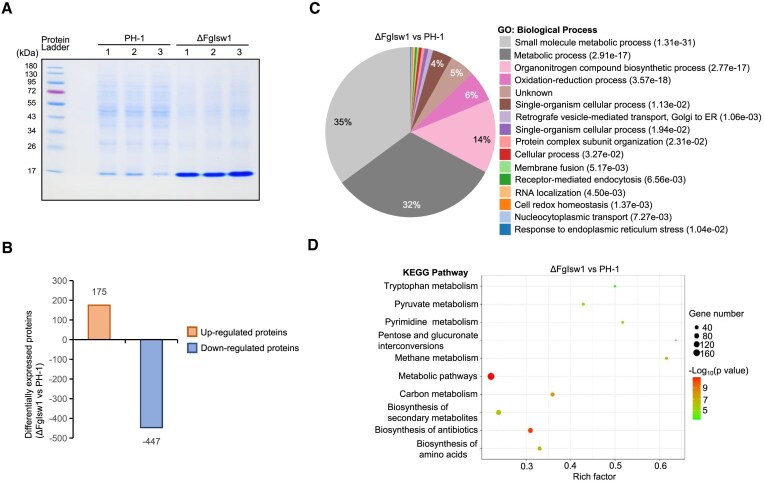
FgIsw1 controls protein translation. (**A**) Protein profiles of the wild-type PH-1 and ΔFgIsw1. Total proteins extracted from each strain were firstly dispersed with SDS–PAGE electrophoresis, followed by coomassie blue staining. The experiment was repeated three times independently with similar results. (**B**) Histograms of up-regulated and down-regulated proteins of putrescine induced ΔFgIsw1 compared with PH-1 based on proteomic data. (**C**) Gene Ontology (GO) classification of biological process of these up- or down-regulated proteins in Fig. 4B. (**D**) Kyoto Encyclopedia of Genes and Genomes (KEGG) pathway analysis of these up- or down-regulated proteins in Fig. 4B.

Given that FgIsw1 regulates tDNAs’ transcription by controlling nucleosome occupancy. To investigate whether FgIsw1 is involved in protein biosynthesis, we compared the total protein profiles of PH-1 and ΔFgIsw1 strains using SDS–PAGE (polyacrylamide gel electrophoresis) electrophoresis followed by coomassie blue staining. The protein profiles differed between the two strains, despite the loaded quantity of each sample was comparable (Fig. 4A). It was highly obvious that ΔFgIsw1 was weaker in most protein syntheses, but it had a much stronger band around 15 kDa than the wild-type PH-1 (Fig. 4A). Mass spectrometric analysis revealed this band represented a set of proteins, mostly weighing 10–20 kDa, including 58 ribosomal proteins (Supplementary Table S6). These findings suggest that FgIsw1 depletion alters ribosomal protein synthesis in *F. graminearum*.

To further characterize the roles of FgIsw1 in protein biosynthesis, we conducted a label-free LC-MS-based quantitative proteomic analysis of PH-1 and ΔFgIsw1 grown in TBI for 40 h. A total of 1843 proteins were detected, with 901 co-expressed in both strains (Supplementary Table S7). Compared to PH-1, ΔFgIsw1 exhibited 622 differentially expressed proteins, with 175 up-regulated and 447 down-regulated, respectively (Fig. 4B). Interestingly, only 16 (9.14%) of the up-regulated proteins showed increased mRNA levels, and 39 (8.72%) of the down-regulated proteins had decreased mRNA levels in ΔFgIsw1.

GO classification and enrichment analysis of the differentially expressed proteins in ΔFgIsw1 and PH-1 revealed major involvement in biological processes such as small molecule metabolic processes (35%), general metabolic processes (31%), and organonitrogen compound biosynthesis (14%) (Fig. 4C and Supplementary Fig. S5A). KEGG pathway analysis indicated that these proteins were primarily involved in the biosynthesis of amino acids, SM, and various metabolic pathways, including carbohydrate, energy, lipid, nucleotide, and amino acid metabolism (Fig. 4D and Supplementary Fig. S5B). Overall, FgIsw1 is essential for balancing the tRNA pool, which in turn affects translation and protein synthesis.

### FgIsw1 modulates DON biosynthesis in a translation-dependent way

**Figure 5.**
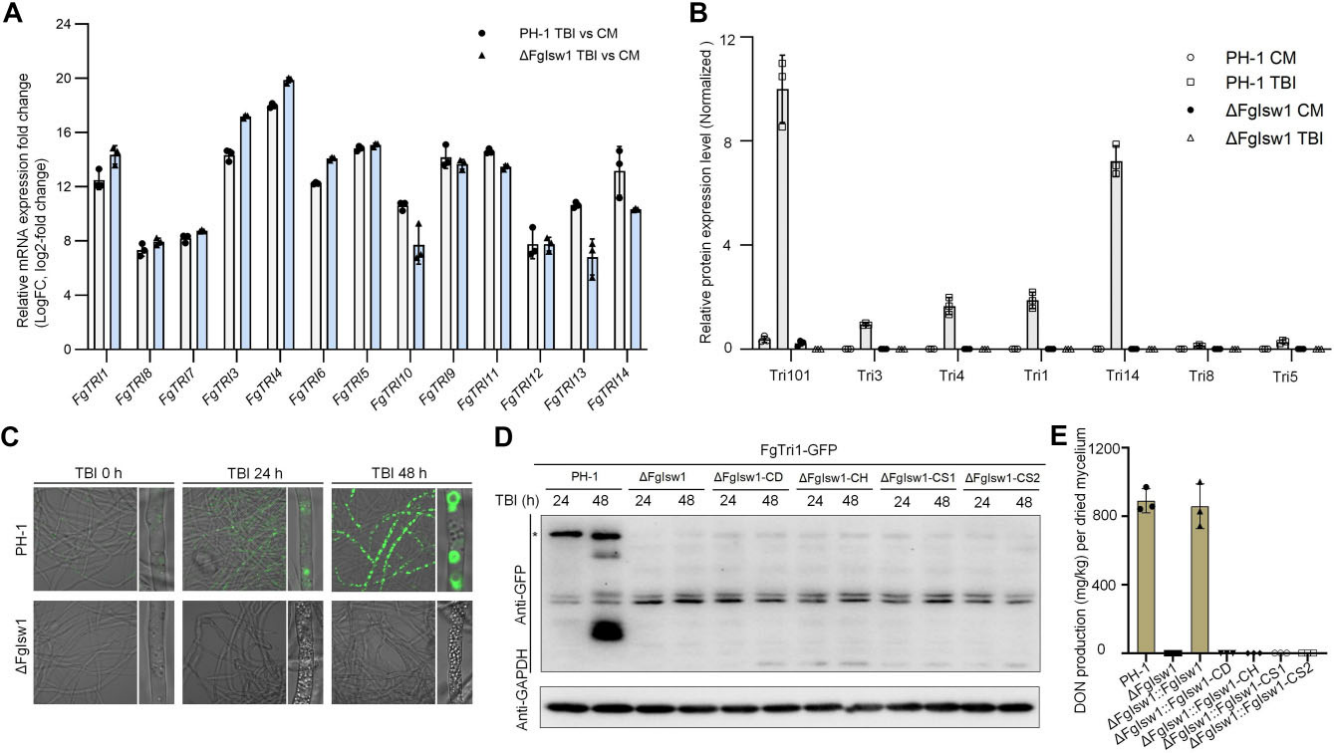
FgIsw1 regulates the expression of Tri proteins and modulates DON biosynthesis. (**A**) Relative expression of DON biosynthetic Tri genes in PH-1 and ΔFgIsw1 grown in TBI based on the transcriptomic data. Data presented are the mean ± standard errors from three repeated experiments. (**B**) Protein levels of these Tri in PH-1 or ΔFgIsw1 grown TBI or CM (complement medium) medium. Data were obtained according to proteomic analysis and presented with the mean ± standard errors from three repeated experiments. (**C**) Toxisome formation of PH-1 and ΔFgIsw1 grown in TBI at different time points. (**D**) FgTri1-GFP protein level detected in PH-1, ΔFgIsw1, and strains with different truncation derivate of FgIsw1 cultured in TBI. The intensity of FgTri1-GFP was determined by immunoblot assay using the anti-GFP antibody. GAPDH (glyceraldehyde-3-phosphate dehydrogenase) was used as the loading control. The experiment was repeated twice independently with similar results. (**E**) DON production of different strains growth in TBI detected with ELISA kit. Each strain was determined for DON production after grown in TBI for 7 days. Data presented are the mean ± standard errors from three repeated experiments.

Given that the FgIsw1 is involved in tRNA transcription and protein synthesis regulation in *F. graminearum*. To confirm the functions of FgIsw1 in DON production, we analyzed its roles in Tri transcription, translation, and measured the final amount of DON of PH-1 and ΔFgIsw1 growth in TBI. As expected, FgIsw1 depletion did not block the inductive expression of Tri genes. After 40 h of incubation in TBI, both ΔFgIsw1 and PH-1 exhibited comparable fold changes in Tri gene expression (Fig. 5A and Supplementary Table S4). However, when fused the ORF of GFP (green fluorescence protein) on the C terminal of Tri1 gene, one of the DON biosynthesis essential gene and used as a marker of re-constructed ER (endoplasmic reticulum)-origin “toxisome” [44, 45], we observed significant differences between the PH-1 and ΔFgIsw1. In the wild-type PH-1 growth in TBI, the fused Tri1-GFP was observed easily either with immunoblotting or confocal microscopy, whereas the translation of Tri1-GFP was undetectable under the same conditions in ΔFgIsw1 (Fig. 5B and C). Furthermore, quantitative proteomics of the ΔFgIsw1 strain identified no peptides from FgTri1 or other Tri proteins, including FgTri3, FgTri4, FgTri5, FgTri8, FgTri11, FgTri14, and FgTri101 (Supplementary Table S8).

In addition, the strains completed with different truncation derivates of FgIsw1, each containing a deletion in functional domain (two chromatin-binding SANT domains, two helicase ATP-binding motifs DEXDc and HELICc as shown in Supplementary Fig. S1A), exhibited similar Tri1-GFP expression deficiencies as ΔFgIsw1 (Fig. 5D). Furthermore, DON productions were consistent with the translation levels of FgTri1-GFP in these strains (Fig. 5E). Taking together, these results indicate that FgIsw1 modulates DON biosynthesis by regulating translation of DON biosynthesis enzymes, not transcription of Tri genes.

### FgIsw1 is required for *F. graminearum* vegetative growth and full virulence

**Figure 6.**
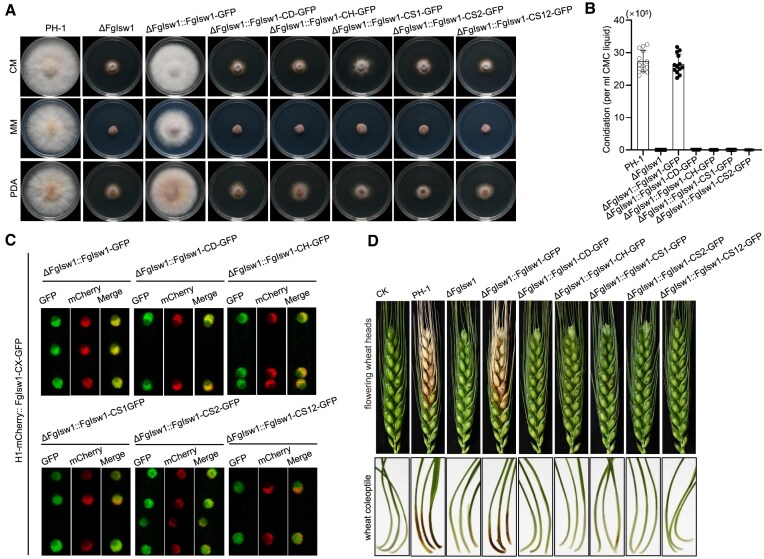
FgIsw1 regulates the development and virulence in *F. graminearum*. (**A**) Mycelial growth of each strain on CM, MM, and PDA plates. FgIsw1 depletion or functional motif (SANT, DEXDc, or HELICc) truncation restrained vegetative growth of *F. graminearum*. (**B**) Histograms of asexual spore production of indicating each strain cultured in CMC broth. Data were presented with the mean ± standard errors from three repeated experiments. (**C**) Functional motif (SANT, DEXDc, or HELICc) truncations had no effect on the subcellular localization of FgIsw1. The wild type or truncated FgIsw1 was fused with GFP and transferred into the ΔFgIsw1 strain in which the H1 (histone) was labeled with mCherry, then the putative co-localization H1 and each FgIsw1 truncation derivate was observed with confocal microscopy. (**D**) The SANT, DEXDc, or HELICc motifs of FgIsw1 were essential for the virulence of *F. graminearum*. Virulence of each strain on flowering wheat was presented as the upper panel, the lower panel was pathogenicity test of each strain on wheat coleoptile.

To analysis the influence of FgIsw1 on fungal growth and differentiation, we characterized ΔFgIsw1 along with four truncation derivatives: ΔFgIsw1::FgIsw1-CD-GFP, ΔFgIsw1::FgIsw1-CH-GFP, ΔFgIsw1::FgIsw1-CS1-GFP, ΔFgIsw1::FgIsw1-CS2-GFP, and the combined mutant ΔFgIsw1::FgIsw1-CS12-GFP. The wild-type PH-1 and wild-type FgIsw1 complemented strain (ΔFgIsw1::FgIsw1-GFP) served as controls. All tested mutants exhibited impaired hyphal growth. Compared to control strains, these mutants formed fewer velvety aerial hyphae and more compact colonies on the agar surface (Fig. 6A). Additionally, mutant colonies were significantly smaller, indicating that FgIsw1 mutations result in a slower hyphal growth rate, and none of these mutants produced conidia after 4 days of incubation at 25°C in sodium carboxymethyl cellulose (CMC) broth (Fig. 6B), whereas each GFP-fused mutated protein localized stably in the nucleus, consistent with wild-type FgIsw1-GFP (Fig. 6C).

Given that DON is a crucial virulence factor for this pathogen, we also assessed the role of FgIsw1 in pathogenicity. Since ΔFgIsw1 and the four truncation derivatives produced no conidia, pathogenicity was tested by point inoculation of wheat heads at anthesis with mycelia. Fifteen days post-inoculati, typical symptoms were observed in the control strains, but ΔFgIsw1, ΔFgIsw1::FgIsw1-CD-GFP, ΔFgIsw1::FgIsw1-CH-GFP, ΔFgIsw1::FgIsw1-CS1-GFP, ΔFgIsw1::FgIsw1-CS2-GFP, and ΔFgIsw1::FgIsw1-CS12-GFP were non-pathogenic on wheat (Fig. 6D, top panel). In addition, the pathogenicity assay was also performed on wheat coleoptile, which was coincided with that on wheat head (Fig. 6D, bottom panel). These results mean that deletion FgIsw1 destroys virulence of *F. graminearum*.

## Discussion

Isw1 orthologues participate in multiple distinct ISWI sub-complexes, including CERC2-containing remodeling complex (CERF), ATP-utilizing chromatin assembly factor (ACF), CHRAC, nucleolar remodeling complex, Williams syndrome transcription factor-ISWI chromatin-remodeling complex (WICH), and remodeling and spacing factor [46–50]. Genetic studies using gene deletion or small interfering RNA (siRNA) inhibition have implicated Isw1 in the control of multiple processes including DNA replication, DNA repair and transcription, chromatin accessibility, as well as nucleosome spacing, and even higher-order chromatin looping [41, 51–55]. Unlike the related SWI/SNF chromatin-remodeling complexes, the ISWI complex cannot evict nucleosomes, rather it slides them to maintain appropriately spaced nucleosome arrays [52, 56–58]. However, until now, no chromatin remodeler such as SWI/SNF, ISWI, INO80, or CHD has been identified that affects translation without impacting target mRNA transcription. Here, we characterized the ISWI chromatin remodeling enzyme FgIsw1 in the phytopathogenic fungus *F. graminearum*, which mediates putrescine-inducible protein translation without significantly influencing mRNA transcripts (Fig. 5, Supplementary Fig. S5, and Supplementary Tables S5 and S6). We found for that this chromatin remodeler FgIsw1 regulates protein biosynthesis by controlling tRNA transcription.

Polymerase III dependent tRNA transcripts may be regulated by the chromatin structure and nucleosome dynamics [59]. The tDNA transcripts are commonly short, nucleosome-free, and have intra-genic promoters [60]. In current study, we found that *FGSG_20508* tDNA was nucleosome-free in the wild type strain. However, the absence of FgIsw1 led to nucleosome retention in its transcription regions, subsequently inhibiting tDNA transcription. At the same time, the nucleosome occupancy of tDNA *FGSG_20660* in the wild type and FgIsw1 depletion strains were contrary, which were consistent with their transcription in different strains respectively (Figs 2 and 3). The primary role of tRNAs is carrying amino acids to ribosomes during translation [61]. It has been shown that the decoding time was closely correlated to tRNA concentrations [62], and cells could alter their tRNA abundance to selectively affect the translation rates of specific transcripts to increase the amounts of required proteins under diverse stress conditions [63, 64]. In line with these, our study found the ISWI chromatin remodeling enzyme Isw1 directly controlled the transcription of tRNA genes. Deletion of Isw1 resulted to a dramatically different expression profile of tRNA genes (Fig. 3), thereby impeding protein translation. Thus, we propose that FgIsw1 regulates homeostasis of tRNA transcription in *F. graminearum* by maintaining nucleosome phasing within chromatin segments of tDNA. When FgIsw1 is deleted, nucleosomes from adjacent regions might slide into or out of tDNA segments, which are typically 76–90 bp in length, subsequently disturbing tDNA transcription. Such nucleosome sliding may have little effect on the transcription of many protein-coding genes, because which are much longer than tRNA genes.

As the primary site of protein synthesis, ribosomes control the rate of protein synthesis according to their amounts [65]. Comparatively, the substrates consumed in translation also function as signals that regulate the synthesis of ribosomal components [66]. Although FgIsw1 deletion led to an overall decrease in protein synthesis (Fig. 4), it should be noted that the expression of some ribosomal proteins increased dramatically (Fig. 4 and Supplementary Table S6). Consistently, Pontes and colleagues found that low cytosolic Mg^2+^ promoted the expression of proteins that import Mg^2+^ in *Salmonella*, but the mutants defective in Mg^2+^ uptake accumulated non-functional ribosomal components and underwent translational arrest [67]. Our results together with these previous findings indicate that the synthesis of tRNAs and proteins could in turn regulate the translation of some ribosomal proteins.

SMs are derived from central metabolic pathways and primary metabolite pools, with acetyl-CoA being the critical initiator of polyketide synthesis and terpene SMs, and amino acids being used for the synthesis of non-ribosomal peptide SMs [68]. Organisms balance their primary and secondary metabolisms according to developmental stages and living environments; the former is indispensable for survival, while the latter is not always so [68, 69]. Farnesyl pyrophosphate, a primary metabolite in *F. graminearum*, is the precursor for trichothecene biosynthesis as well as ergosterol [70]. However, treatment of *F. graminearum* with certain concentrations of ergosterol biosynthesis inhibitors inhibited mycelial growth but promoted DON production [20]. Environmental stimuli can either activate or repress secondary metabolisms since they must maintain dynamic homeostasis with related primary metabolic pathways but are interdependent with others. Here, we found that FgIsw1 modulated the transcription of tRNAs, regulating protein synthesis and DON biosynthesis under putrescine induction conditions. Given the universal functions of tRNAs, it is intriguing why DON biosynthetic proteins were selectively repressed completely, while general proteins were not (Figs 4 and 5). Considering the DON-inducing TBI medium is nutrient deficient with putrescine as the only nitrogen source, it is reasonable to propose that the ΔFgIsw1 mutant prioritized survival over secondary DON biosynthesis under nutrition stress conditions.

The mechanisms of DON biosynthesis and its regulation are crucial for FHB control. Previous studies on DON biosynthesis enzymes have focused on the transcriptional regulation of DON biosynthesis genes [20, 69]. Here, we found that the chromatin remodeling factor FgIsw1 is involved in DON biosynthesis regulation by maintaining the nucleosome-free status of tRNA segments and controlling tRNA transcription (Fig. 2). The abundance of tRNA guarantees the translation of Tri proteins when the fungus is grown in TBI broth. Given the importance of this mycotoxin in fungal virulence, manipulating FgIsw1 (e.g. RNAi or host-induced gene silencing) may represent a promising strategy for managing DON mycotoxin and FHB, a possibility that merits further study. This novel insight could lead to new strategies for disease and mycotoxin control in the field.

## Supplementary Material

gkaf225_Supplemental_Files

## Data Availability

Raw reads of ChIP-seq, ATAC-seq, and RNA-seq generated in this study have been deposited in the National Genomics Data Center under project accession code PRJCA028651. Data have been deposited in the Genome Sequence Archive in National Genomics Data Center, Beijing Institute of Genomics (BIG), Chinese Academy of Sciences, under accession number CRA18119, CRA018151 and CRA018665. All accession numbers are included in the BioProject PRJCA028651. UCSC genome browser session displaying the ChIP-seq tracks: https://genome.ucsc.edu/s/Jing%20W/ChIP%2Dseq%20analysis%20of%20FgIsw1. The mass spectrometry-based proteomics data from this study have been submitted to ProteomeXchange under the accession code PXD055879.
