# Supplementary material for: Fungal chromatin remodeler Isw1 modulates translation via regulating tRNA transcription
Source: Nucleic Acids Res. 2025 Apr 1;53(6):gkaf225. doi: 10.1093/nar/gkaf225 (PMC11959538; doi:10.1093/nar/gkaf225)
Supplement: gkaf225_Supplemental_Files [file gkaf225_supplemental_files.zip › ISW1 Supplementary Figures.pdf]

Fungal chromatin remodeler Isw1 modulates translation via regulating tRNA transcription

Jing Wang et al.

Supplementary Figures

Figure S1

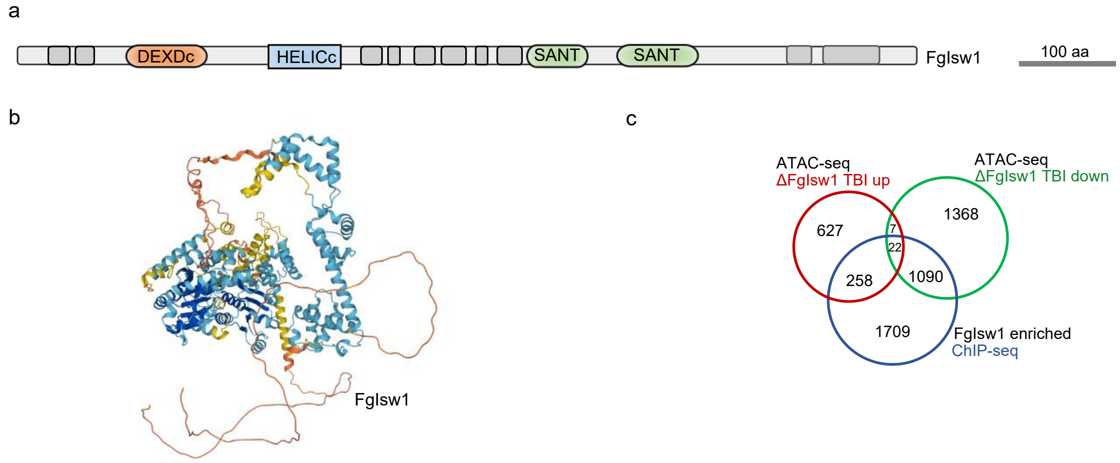

Figure S1. FgIsw1 has chromatin binding activity. a. The schematic structure of FgIsw1. FgIsw1 has a DEXDc Helicase ATP-binding motif, a HELICc Helicase ATP-binding motif, and two SANT chromatin binding motifs. **b**. Prediction of secondary structure of FgIsw1 captured from Alphafold (https://alphafold.ebi.ac.uk/entry/I1S0N5). **c**. Venn diagram showing the overlap between the genes corresponding to FgIsw1 enriched peaks, ΔFgIsw1 ATAC-seq peaks (both decreased and increased) compared with PH-1.

Figure S2

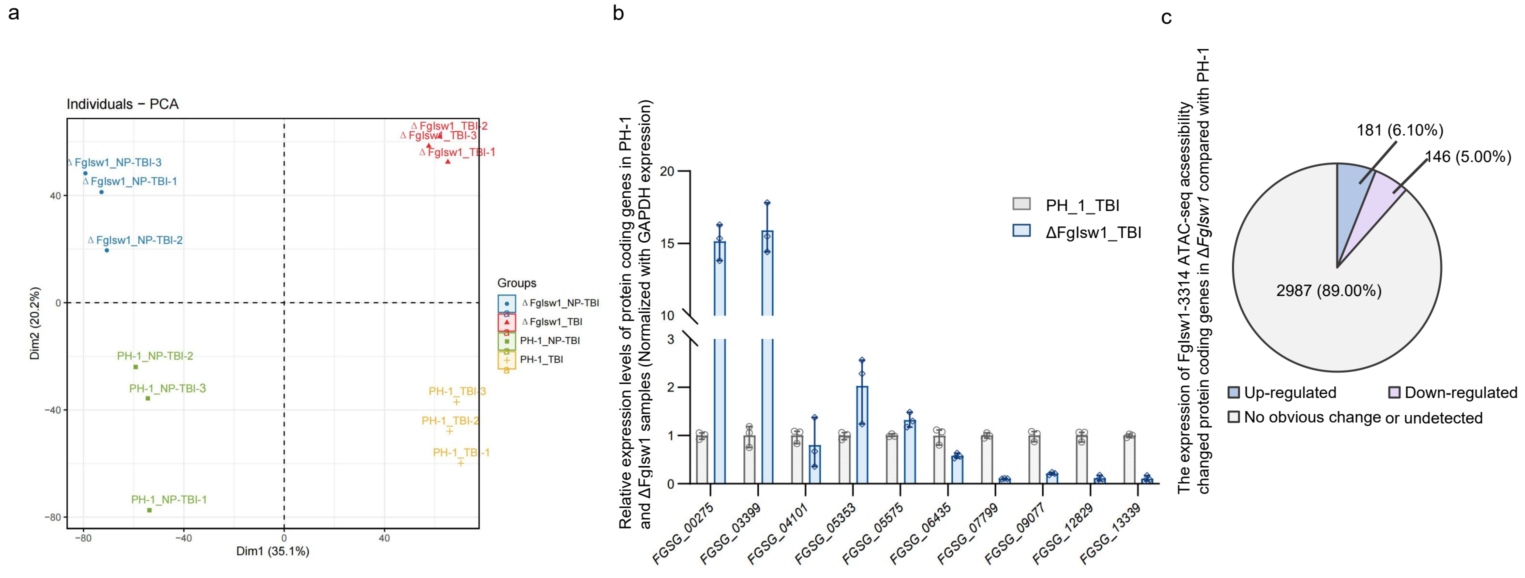

Figure S2. FgIsw1 binds tDNAs and modulates tRNAs' abundance. a. Factor map of the PCA (principal component analysis) performed on 12 RNA-seq samples in this study. Four cluster groups were identified, TBI cultured ΔFgIsw1 (red), NP-TBI cultured ΔFgIsw1 (blue), TBI cultured PH-1 (yellow), and NP-TBI cultured PH-1 (green), indicating that there are good correlations between replicates in one group. **b**. RT-qPCR determination of relative gene expression levels in TBI cultured PH-1 and ΔFgIsw1 (n=3). **c**.FgIsw1 binds protein coding genes but doesn't affect their transcription. Pie chart of expression change of FgIsw1 enriched protein coding genes caused by FgIsw1 deletion.

Figure S3

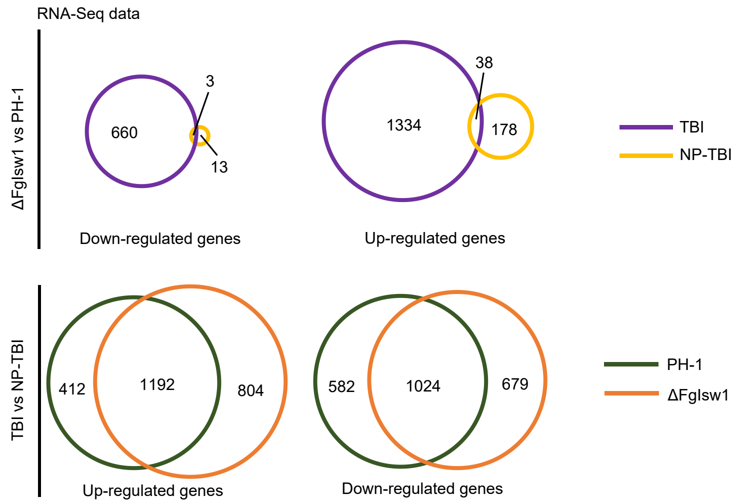

Figure S3. Venn diagrams showing the overlap between the FgIsw1 depletion resulted to up-regulated or down-regulated gene expression under different conditions. The upper panel shows that mild gene expression changes between ΔFgIsw1 and the wild type PH-1 grown in NP-TBI condition, but more pronounced changes when the strains were grown in TBI. The bottom panel shows that there were high overlaps of the expression patterns between the wild type PH-1 and the ΔFgIsw1 grown in TBI.

Figure S4

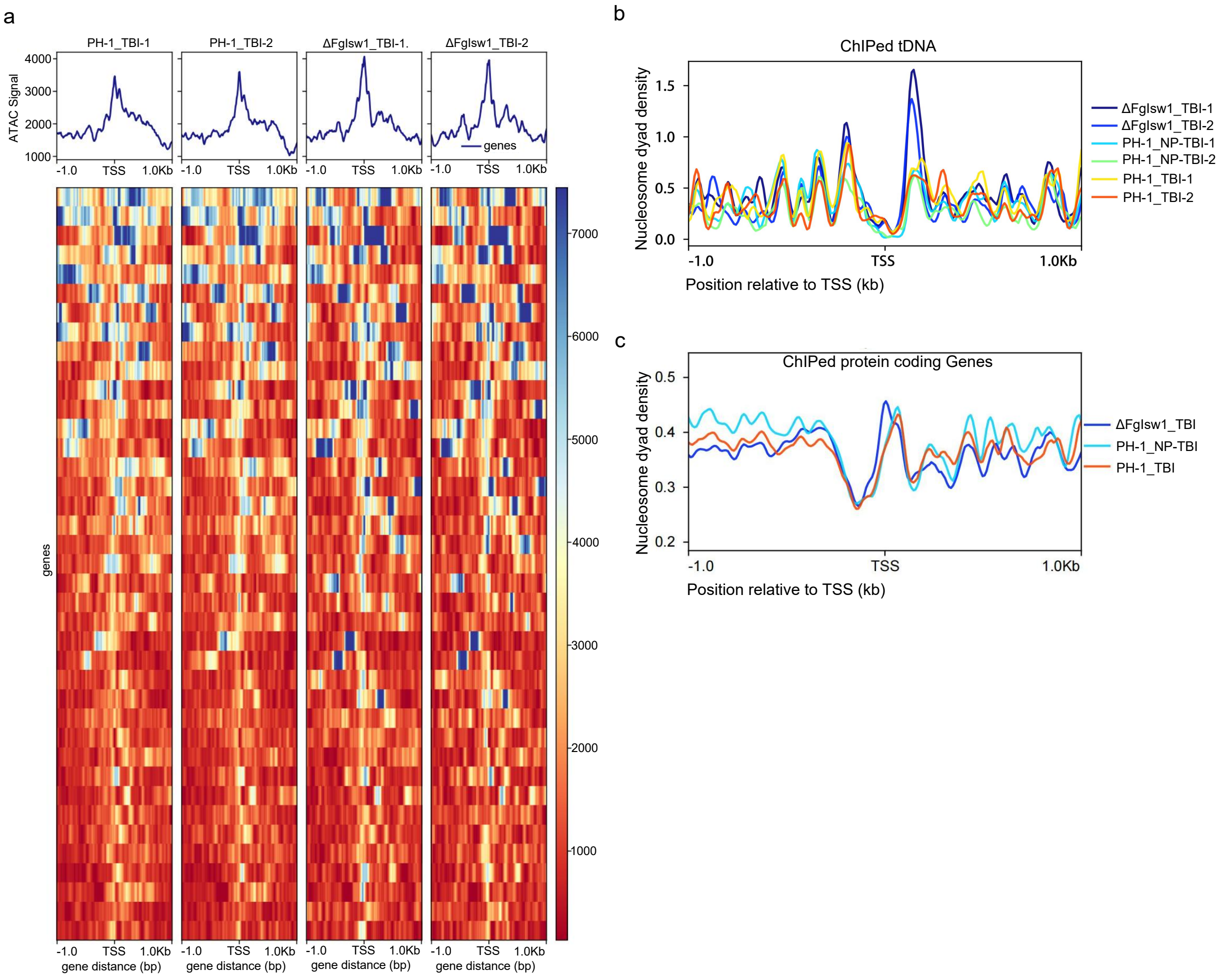

Figure S4. FgIsw1 affects nucleosome occupancy of tDNAs. a. The accessibilities of FgIsw1-enriched tDNAs in PH-1 and ΔFgIsw1 grown in TBI. **b**. Histograms of nucleosome occupancy plotted based on ATAC-seq data around all FgIsw1 enriched tDNAs from the ChIP-Seq data. The signal was plotted ±1 kp from the TSS, different samples including ΔFgIsw1 and wild type PH-1 grown in TBI (PH-1_TBI and ΔFgIsw1-TBI), and in non-putrescine containing TBI (PH-1_NP-TBI) were showed with different colors. **c**. Histograms of nucleosome occupancy plotted based on ATAC-seq data around all FgIsw1 enriched protein coding genes in these three samples. The signal was plotted ±1 kp from the TSS.

Figure S5

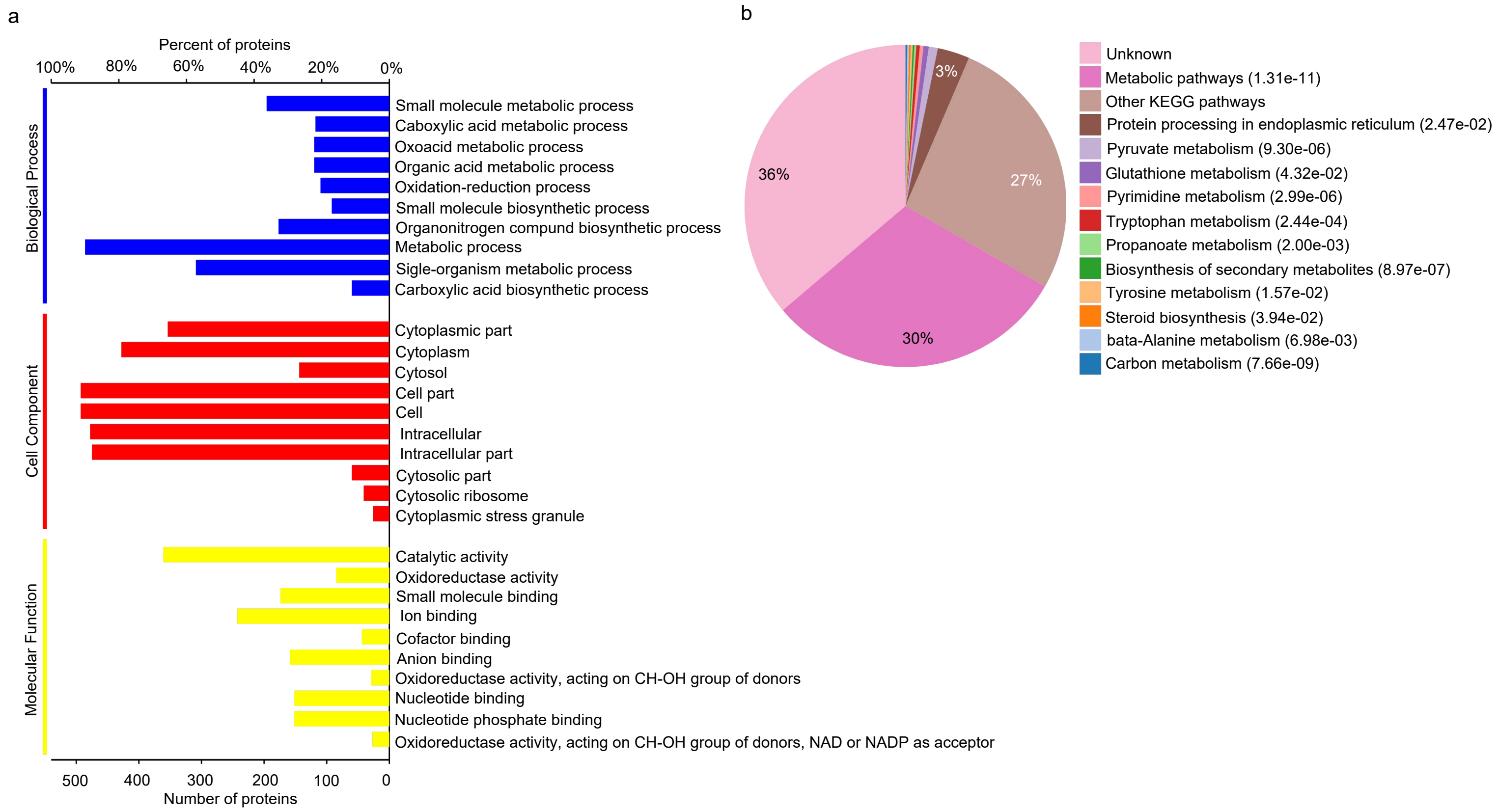

Figure S5. FgIsw1 depletion causes global protein expression alteration. a. The GO (Gene Ontology) classification and enrichment analysis of differentially expressed proteins in ΔFgIsw1 compared with the wild type PH-1 grown in TBI. **b**. The KEGG (Kyoto Encyclopedia of Genes and Genomes) pathway analysis of the differential expressed proteins ΔFgIsw1 compared with the wild type PH-1 grown in TBI.
